# Nutcracker Syndrome: A Cause of Hematuria and Low Back Pain in Young Patients

**DOI:** 10.7759/cureus.31290

**Published:** 2022-11-09

**Authors:** Sara Santos, Luís Fernandes, Sofia Ferreira, Alexandra Reis, Alcinda M Reis

**Affiliations:** 1 Internal Medicine, Centro Hospitalar de Entre Douro e Vouga, Santa Maria da Feira, PRT; 2 Radiology, Centro Hospitalar de Entre Douro e Vouga, Santa Maria da Feira, PRT

**Keywords:** hematuria, young patients, anemia, low back pain, nutcracker syndrome

## Abstract

Nutcracker syndrome is a clinical condition in which there is compression of the left renal vein in its path between the abdominal aorta and the superior mesenteric artery. This phenomenon can cause abdominal or low back pain and hematuria. It is a rare clinical entity, although probably underdiagnosed. The diagnosis is essentially clinical and based on imaging, but necessarily a diagnosis of exclusion.

We present the case of a 21-year-old boy who came to the Emergency Department with hematuria and low back pain. After exhaustive study and exclusion of other possible clinical entities, the diagnosis was confirmed to be nutcracker syndrome. Despite its usually benign expression, this entity should not be forgotten in the diagnostic process of cases of hematuria and low back pain, especially in young patients.

## Introduction

Nutcracker syndrome (NCS) is a rare clinical condition caused by compression of the left renal vein (LRV) by the superior mesenteric artery (SMA) as it passes between the SMA and the abdominal aorta (AA) (anterior NCS) [1]. Another variation of this entity, known as posterior NCS, occurs when the LRV is retroaortic and becomes compressed between the spine and the AA [1].

The extrinsic stenosis of the renal branch can cause different symptoms, with the most common being the absence of symptomatology; however, it can cause macroscopic hematuria, proteinuria, renovascular hypertension, flank pain, dyspareunia, dysmenorrhea, and pelvic varicose veins [1].

There appears to be a bimodal distribution of cases. Most patients with complaints of hematuria and low back pain are teenagers or young adults who tend to be slender. The second peak incidence is in middle-aged women who present mostly with pelvic congestion syndromes [2].

Diagnosis is challenging and is usually made after excluding other more common causes as there are no specific clinical diagnostic criteria [1].

Imaging examinations are necessary to confirm the syndrome, and Doppler ultrasonography is the most widely used method [1-3]. Treatment varies according to the severity of symptoms, ranging from conservative management for young patients or those with mild symptoms to surgical and endovascular approaches for those who do not improve after conservative management or who have severe symptoms [4].

## Case presentation

A 21-year-old male, with no relevant previous pathological history and no usual medications, presented to the Emergency Department for lower back pain with one week of evolution and macroscopic hematuria with one day of evolution. He denied dysuria, pollakiuria, abdominal pain, nausea, vomiting, fever, and a history of recent trauma or vigorous exercise. He also reported that three years earlier he had an episode of mild macroscopic hematuria with spontaneous resolution, which did not motivate the use of health services.

Physical examination revealed a bilaterally negative renal Murphy’s sign and no other changes. His blood pressure was normal. Analytical results showed mild microcytic and hypochromic anemia and no increase in acute inflammatory markers (Table 1). Type II urine test showed 25 leukocytes, negative nitrites, absence of albuminuria and proteinuria, and 25/µL erythrocytes without dysmorphia. The urine culture test was negative.

**Table 1:** Analytic results.

Parameter	Value	Reference value
Hemoglobin (g/dL)	11.2	12.0–16.0
Hematocrit (%)	34.5	36.0–46.0
Leukocytes (×10^9^/L)	6.6	4.0–11.0
Platelets (×10^9^/L)	187	150–450
International normalized ratio	1.0	1.0
Troponin (ng/L)	<1.6	≤34.0
Myoglobin (ng/mL)	16.4	<140
Lactate dehydrogenase (U/L)	186	125–220
Creatinine (mg/dL)	1.0	0-6–1.1
Blood urea nitrogen (mg/dL)	34	21–43
Albumin (g/dL)	4.1	3.5–5.0

Ultrasonography showed no morphological changes in the kidneys or bladder.

He was admitted to the Internal Medicine ward and started analgesic treatment with resolution of hematuria and low back pain in three days. Diagnostic investigation excluded hereditary thrombophilia, hyperhomocysteinemia, and antiphospholipid syndrome.

**Figure 1:**
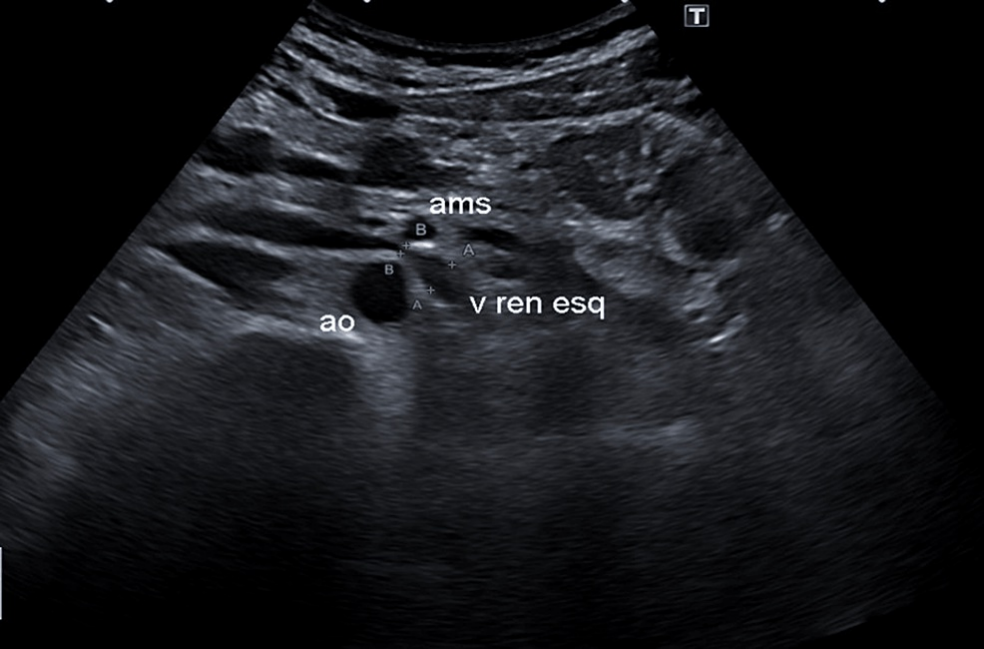
Doppler ultrasonography showing changes in the diameter of LRV between the AA and the SMA suggestive of NCS. LRV: left renal vein; AA: abdominal aorta; SMA: superior mesenteric artery; NCS: nutcracker syndrome

Doppler ultrasonography showed a change in the caliber of the LRV between the aorta and the SMA after the origin (relation between the diameter upstream of the compression (6.8 mm) and in the compression (2.9 mm): 2:35), suggestive of NCS (Figure 1).

**Figure 2:**
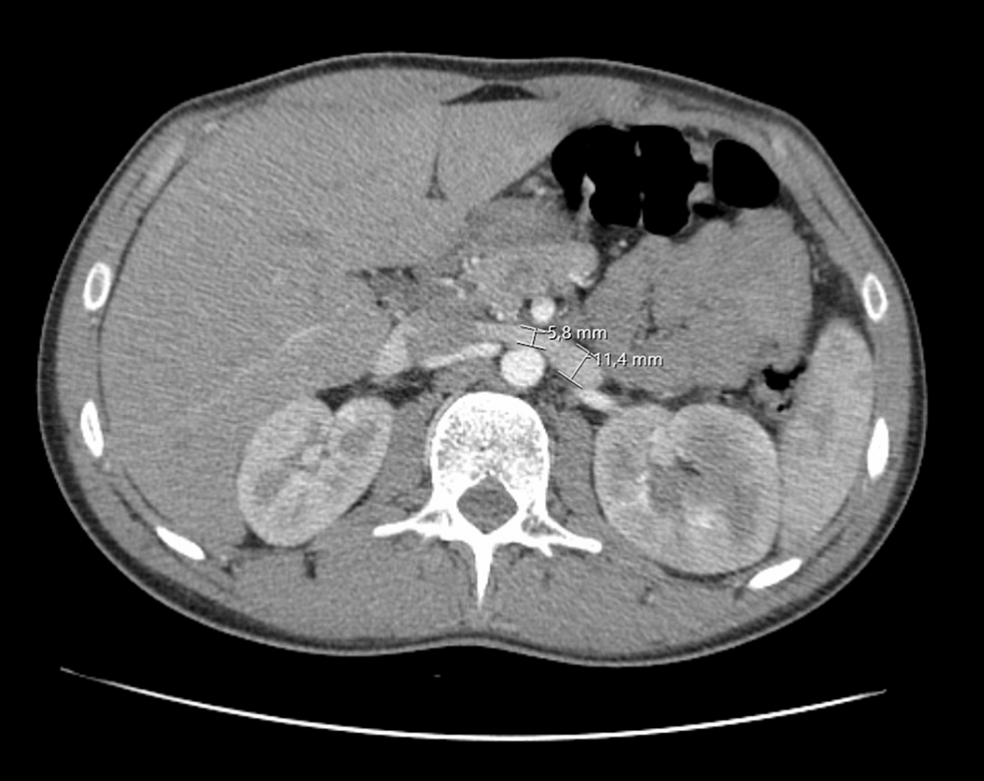
CT angiography followed by an acquisition in the excretory phase (CT urography) showing compression of the LRV in its path between the AA and SMA. CT: computerized tomography; LRV: left renal vein; AA: abdominal aorta; SMA: superior mesenteric artery

**Figure 3:**
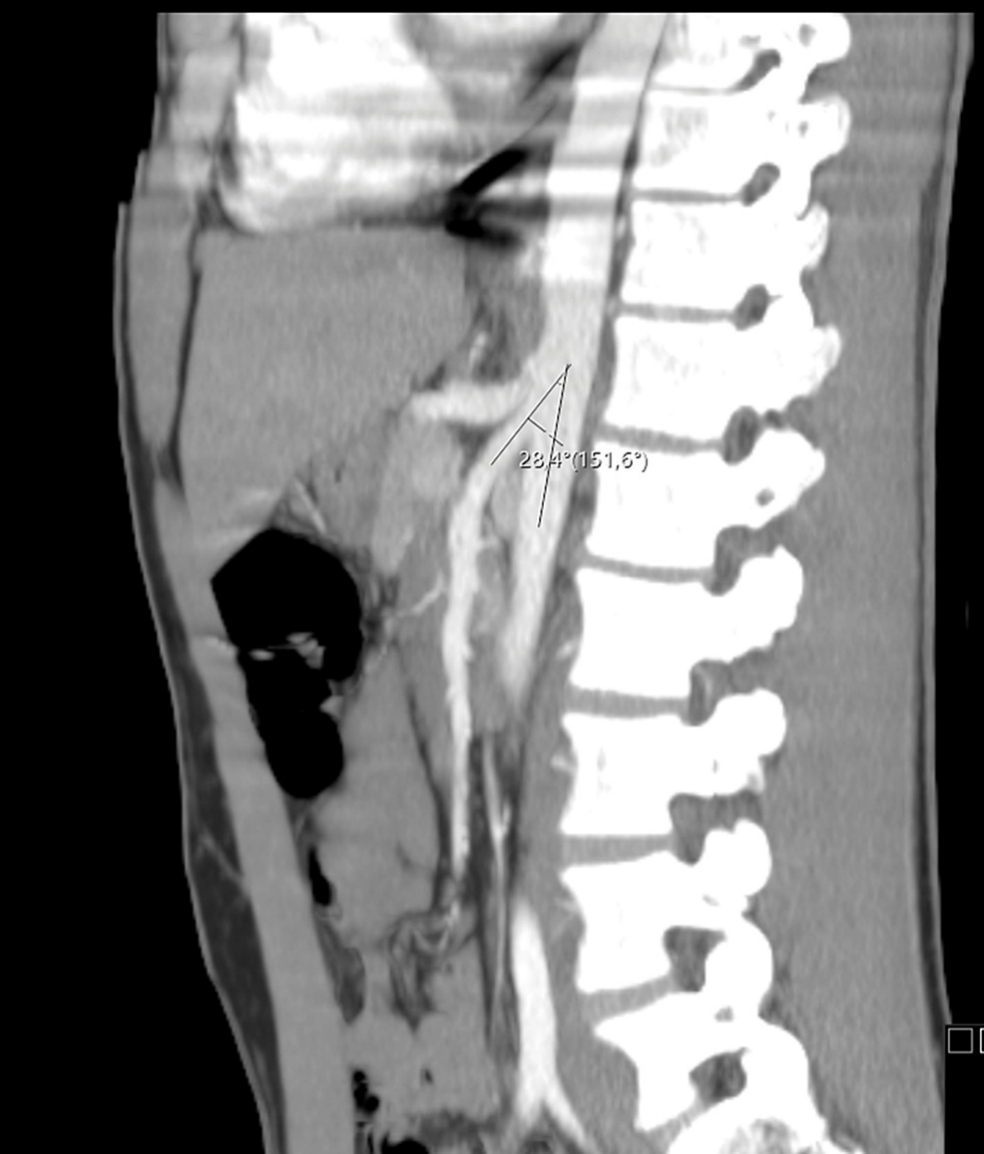
CT angiography followed by an acquisition in the excretory phase (CT urography) showing an angle between the SMA and aorta of 28.4°. CT: computerized tomography; SMA: superior mesenteric artery

Once all other potential causes of hematuria and low back pain were excluded and Doppler ultrasonography findings were suggestive of NCS, a computerized tomography (CT) urogram and CT angiography were performed to confirm the diagnosis. CT urogram and CT angiography showed compression of the LRV in its path between the AA and SMA (Figure 2) and an angle between the SMA and aorta of 28.4° (Figure 3), which confirmed the diagnosis of NCS.

The patient was evaluated by the Urology team and was discharged with conservative treatment. Two months after this episode, he was observed at an outpatient clinic, remaining asymptomatic, without new episodes of hematuria and low back pain.

## Discussion

The clinical presentation of NCS ranges from asymptomatic in most cases to various symptoms, including hematuria and abdominal pain [5]. Hematuria, as seen in this case, results from increased blood pressure of the LRV, resulting in rupture of the thin-walled septum between the varicose veins and the collecting system of the renal fornix [5]. The pain is a result of the inflammatory cascade triggered by venous hypertension [5].

The diagnosis is hard and requires excluding other more common causes. Confirmation of the diagnosis requires imaging examinations. Doppler ultrasonography is the first-line diagnostic test with 69-90% sensitivity and 89-100% specificity [6]. The suggestive findings on Doppler ultrasonography are a five-fold decrease in the anteroposterior diameter of the LRV in the aorto-mesenteric passage in relation to the renal hilum, as well as a five-fold increase in peak velocity between the aortomesenteric passage and the hilum kidney [7]. Although it is recommended as the first diagnostic investigation, its index and range of values in the diagnosis of NCS are highly variable [8]. This is because findings vary depending on whether the patient’s position is supine, prone, or upright, as well as technical difficulties arising from the very small sampling area [8].

When Doppler ultrasonography is elucidative and the symptoms are mild, CT angiography is not essential. Important findings demonstrated by CT angiography are the Beak sign, a severe form of narrowing of the LRV at the aortomesenteric portion, and an angle between the SMA and the aorta <41° [9].

Treatment can be conservative, surgical, medical, or endovascular. Conservative treatment is suggested for patients with tolerable symptoms, such as mild hematuria; surgical treatment is indicated in cases of severe haematuria associated with anemia, functional renal failure, severe pelvic pain, or ineffectiveness of conservative treatment after 24 months of clinical follow-up [10].

## Conclusions

NCS is a rare clinical condition that should be considered in patients with left flank or lumbar pain and isolated hematuria. The diagnosis is essentially clinical and based on imaging, but necessarily a diagnosis of exclusion. Doppler ultrasonography is the first-line imaging examination but CT angiography can be used to confirm the diagnosis. Treatment is usually conservative. In this case, knowledge of the syndrome was essential for its suspicion and subsequent confirmation, avoiding performing more invasive procedures.
